# Occurrence of *Staphylococcus aureus* TSST-1 in Foods: A Review

**DOI:** 10.3390/toxins17120606

**Published:** 2025-12-18

**Authors:** Maria Govari, Andreana Pexara

**Affiliations:** 1 Department of Food Science and Technology, School of Agriculture and Food, University of the Peloponnese, 24100 Kalamata, Greece; margochem@gmail.com; 2 Laboratory of Hygiene of Foods of Animal Origin, Faculty of Veterinary Medicine, University of Thessaly, 43100 Karditsa, Greece

**Keywords:** *Staphylococcus aureus* TSST-1, toxic shock syndrome, staphylococcal food poisoning (SFP), *tst* gene, mastitic milk

## Abstract

Toxic Shock Syndrome Toxin-1 (TSST-1) is produced by *Staphylococcus aureus* strains encoded by the *tst* gene. Toxic shock syndrome (TSS) is a severe disease caused by TSST-1 toxin and associated with staphylococcal food poisoning (SFP). The aim of the present review was to present data on the occurrence of *S. aureus* TSST-1 in foods published in various countries. PCR-based assays are most frequently used for the detection of *S. aureus* TSST-1 in foods. *S. aureus* TSST-1 is predominantly detected in foods of animal origin. The highest occurrence has been observed in mastitic ruminants’ milk, indicating that mastitis is a risk of milk contamination with the pathogen. High occurrence rates of *S. aureus* TSST-1 have also been identified in raw milk and artisanal cheeses. Various occurrence levels have also been reported in beef, pork, lamb, and chicken meat. Low occurrence levels have also been reported for fish or other seafood products. The *tst* gene was also found in combination with other toxigenic genes in *S. aureus* TSST-1 isolates (e.g., MRSA or Panton-Valentine Leukocidin, PVL). Monitoring *S. aureus* TSST-1 in food is important for public health because food can be a vehicle for transmitting the antibiotic-resistant pathogen to humans.

## 1. Introduction

*Staphylococcus aureus* is a Gram-positive bacterium found in the commensal microbiota of humans and animals, and it is also an important opportunistic pathogen. Among the most common symptoms of *S. aureus* human infections are bacteremia, toxic epidermal necrolysis, scalded skin syndrome (newborn), skin abscesses, endocarditis, osteomyelitis, septic arthritis, and pneumonia [1]. According to official reports of the European Food Safety Authority (EFSA), the number of *S. aureus* outbreaks in the European Union increased from 114 outbreak cases (records of the years 2014 to 2018) to 177 outbreak cases (records of years 2019 to 2020) [2]. In USA, 75 outbreak cases were recorded from the years 2009 to 2015 [3].

**Figure 1:**
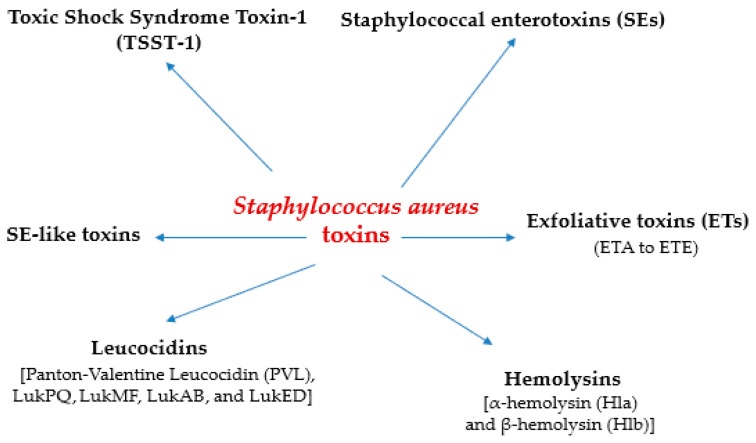
*S. aureus* toxins associated with foods.

*S. aureus* is also a significant food borne pathogen. It is associated with staphylococcal food poisoning (SFP), which is caused by the consumption of foods contaminated with staphylococcal enterotoxins (SEs) produced by *S. aureus* [4]. Among the enterotoxins associated with SFP are staphylococcal enterotoxins (SEs) and SE-like toxins, which present superantigen activity, as well as exfoliative toxins (ETs) (ETA to ETE), which are serine proteases that cause skin exfoliation and blistering by cleaving desmoglein 1 (Dsg1) [5]. The majority of confirmed SFP cases (almost 95%) are attributed to the five classical SEs (SEA to SEE) as compared to other newer identified emetic SEs (SEG-SEJ and SEK–SEX) [6,7,8]. Toxic shock syndrome toxin 1 (TSST-1) is a pyrogenic toxin superantigen [5]. TSST-1 is encoded by the *tst* gene in *S. aureus* DNA and the pathogen is referred to as *S. aureus* TSST-1 [1]. *S. aureus* toxins associated with foods are shown in Figure 1, while activity of *S. aureus* toxins is also displayed in Table 1.

Toxic shock syndrome (TSS) is a severe, life-threatening disease caused by TSST-1 produced primarily by *S. aureus* or *Streptococcus pyogenes* [5]. The disease manifests suddenly, with symptoms that may include nausea, vomiting, headache, diarrhea, redness of the mouth and eyes, confusion, high fever, skin rash, low blood pressure (hypotension), rapid organ failure (particularly of the liver and kidneys), shock, and, in severe cases, death [4]. The TSST-1 has super-antigenic activity and stimulates T-cells, resulting in excessive cytokine production and finally causing various organ failures [9]. Although the incidence rate of TSS is rather low, the TSS treatment is a difficult process in many cases since it requires urgent hospital care with use of broad-spectrum antibiotics to control the infection and intensive care for respiratory difficulties [10]. The burden of *S. aureus* TSST-1 diseases is also associated with the complexity of *S. aureus* virulence factors [1].

In USA, 21,465 TSS patients were recorded in hospitals from 2003 to 2012, with an estimated annual incidence of 6.65 cases per million persons and a crude mortality rate of 9.6% [11]. Sharma et al. [12] reported a TSS rate of 0.07 per 100,000 people, based on summarized data of cases that occurred in the UK between 2008 and 2012. The TSS treatment is also a difficult process when the *S. aureus* TSST-1 microbiome is associated with other virulence or antimicrobial resistance factors, e.g., methicillin-resistant *S. aureus* (MRSA) strains [13]. According to an epidemiological study accomplished in USA (2008–2017), among MSSA (methicillin-sensitive *Staphylococcus aureus*) and MRSA patients the TSST-1 prevalence was 6% [14].

The genes encoding the various SEs are found in different mobile genetic elements (MGEs), such as enterotoxin gene clusters (EGC), plasmids, pathogenicity islands (SaPIs), and the staphylococcal cassette chromosome (SCC) [15]. The *tst* gene in *S. aureus* TSST-1 DNA is usually found in combination with other toxigenic genes [16]. SEs (SEA to SEE, SEG to SER, and SEU) are encoded by *sea* to *see*, *seg* to *ser*, and *seu* genes [5]. SE-like toxins (SEG to SET) are encoded by *seg* to *set* genes [17]. Panton-Valentine Leucocidin (PVL) is a toxin produced by *S. aureus* that damages blood cells and attacks immune cells, and there are other pore-forming leucocidins such as LukPQ, LukMF, LukAB, and LukED [18]. Leucocidins PVL, LukAB, LukED, LukPQ, and LukMF are encoded by the *lukPV*, *lukA*-*lukB*, *lukED*, *lukPQ*, and *lukMF* genes [5]. *S. aureus* α-hemolysin (Hla) and β-hemolysin (Hlb) are pore-forming toxins encoded by the *hla* and *hlb* genes [19]. *S. aureus* exfoliative toxins (ETA-ETE) are serine proteases that cause skin blisters and staphylococcal scalded skin syndrome (SSSS) and are encoded by *eta* to *ete* genes, respectively [20]. *S. aureus* staphylokinase (SAK) is a plasminolytic enzyme that prevents the formation of blood clots in humans and is encoded by the *sak* gene [21]. The *S. aureus icaA* gene is associated with the synthesis of the IcaA protein and contributes to biofilm formation [22]. The MRSA resistance to β-lactam antibiotics is primarily associated with the *mecA* gene [23].

In 1980s, the majority of *S. aureus* TSST-1 infections were of menstrual origin due to contamination with the pathogen of high absorbency tampons or cups [24]. In later years the menstrual TSST-1 infections were almost eradicated due to prophylactic and hygienic measures [25,26]. Foodborne TSST-1 outbreaks have been recorded in recent years, but according to epidemiological data, the incidence is rather low [27]. The presence of *S. aureus* TSST-1 in various foods (e.g., milk, cheese, meat, or shrimp) was also identified [26]. According to our examination of the literature, no review article has been found addressing the occurrence of *S. aureus* TSST-1 in foods.

This review summarizes global data on the prevalence and detection of *S. aureus* TSST-1 in foods of animal and non-animal origin.

## 2. Methodology

This review was conducted through a comprehensive search of the scientific literature to identify studies reporting the occurrence of *S. aureus* TSST-1 in foods. Electronic databases including PubMed/MEDLINE, Scopus, Web of Science, and Google Scholar were searched for publications available until September 2025. Search terms included combinations of keywords such as “*Staphylococcus aureus* TSST-1”, “milk”, “meat”, “foods”, “fish”, “isolation methods, “outbreaks”, “staphylococcal food poisoning (SFP)” “health”, “TSST-1”, “toxic shock syndrome”, “*tst* gene”, “staphylococcal enterotoxins (SEs)” using Boolean operators (AND, OR), and MeSH terminology where applicable.

Eligible sources included original research articles, surveillance reports, food safety assessments, and reviews addressing the detection, prevalence, and molecular characterization of *S. aureus* TSST-1 in various foods. No language restrictions were imposed; however, English-language articles were prioritized when translations were unavailable. Reference lists of retrieved publications were manually screened to identify additional relevant studies.

The selection process prioritized studies that provide insight into the occurrence of *S. aureus* TSST-1 in foods, the molecular characteristics of the *tst* gene, associations with other virulence genes or antimicrobial resistance toxigenic genes such as *mecA* or PVL genes, contamination routes along the food chain, and public health risks. Special attention was given to studies that examined TSST-1 presence in raw milk, the role of mastitis in milk contamination, and the presence of TSST-1-producing strains in dairy products, meat, seafood, and ready-to-eat foods. Data were synthesized narratively, with a focus on integrating molecular and genomic findings with epidemiological patterns, food safety risks, and public health perspectives. Additionally, knowledge gaps relevant to surveillance, prevention, and control strategies for *S. aureus* TSST-1 in foods were identified.

## 3. Isolation and Identification of *S. aureus* TSST-1 in Foods

The isolation and identification of *S. aureus* from food samples relies on a combination of culture-based and confirmatory laboratory methods. Food samples are cultured on selective growth media such as Baird-Parker Agar supplemented with egg yolk tellurite emulsion or Mannitol Salt Agar, which support *S. aureus* growth, and the pathogen is differentiated from other staphylococci species [28,29]. The procedure of isolation on the Baird-Parker Agar is also described by) ISO 6888-1:2021 [30].

Confirmation of the isolates to the species level is based upon Gram staining, catalase reaction, and biochemical test characterization (e.g., API ID 32 Staph, bioMérieux, Marcy l’Etoile, France). Additional plating on Columbia agar with defibrinated sheep blood is also used for hemolysis reaction [31].

Biosensor-based detection systems were also used for the rapid detection of *S. aureus* and TSST-1 in foods [32]. Aptamer and nucleic acid-based detection technologies have recently been used for rapid on-site monitoring of *S. aureus* in foods [33]. The dual recombinase polymerase amplification system was combined with lateral flow immunoassay for simultaneous detection of *S. aureus* and co-contaminants [34].

PCR methods are the most important methods that are mostly used for detecting *S. aureus* TSST-1 in foods [27,35]. Various methods are used for the DNA extraction, but commercial extraction kits (e.g., QIAamp DA minikit, Qiagen, Dusseldorf, Germany) and phenol-chloroform extraction are the most common methods [36]. Primer sequences for the PCR detection of the *tst* gene have also been described in several previous studies [37,38]. Multiplex PCR is also efficient at identifying the *tst* gene with other virulence and toxigenic genes (e.g., *sea* to *see* genes) or resistance genes (*mecA*) [39,40]. The real-time PCR method has also been reported for detecting *S. aureus* TSST-1 in foods [28]. Additionally, MALDI-TOF mass spectrometry has been used to confirm the identification of *S. aureus* TSST-1 [41].

Other methods have also been employed for the detection of S. aureus TSST-1, primarily in clinical isolates. These methods include Whole-Genome Sequencing, latex agglutination, mass spectrometry, and T-cell activation (bioassay) [27,42,43]. Enzyme-Linked Immunosorbent Assays (ELISA) have also been used for the identification of *S. aureus*-harboring classical SEs genes, but not the *tst* gene in foods (e.g., RIDASCREEN^®^ SET Total R-Biopharm AG, Darmstadt, Germany) or immuno-enzymatic tests Vidas^®^ SET2 (bioMérieux, Marcy l’Etoile, France) [28]. TST-RPLA-TD940 (Oxoid, Cheshire, UK) is a reversed passive latex agglutination test used for *S. aureus* TSST-1 [27].

To the best of our knowledge, no study has reported the existence of TSST-1 in foods except in foodborne *S. aureus* TSST-1 outbreak cases. *S. aureus* TSST-1 has been identified in clinical isolates of patients [42]. Τhe following methods for the detection of SEs in foods were reported: bioassay method, molecular methods, e.g., PCR, polymer-based biosensors, aptamers (oligonucleotides RNA or DNA), ELISA, liquid chromatography coupled with high-resolution mass spectrometry (HRMS-LC), and fluorescence sensors [35,44]. An overview of the main detection, isolation, and identification method for *S. aureus* TSST-1 in foods is summarized in Table 2. However, almost all these methods are used for the detection of SEs other than TSST-1.

## 4. Occurrence of *S. aureus* TSST-1 in Foods

**Figure 2:**
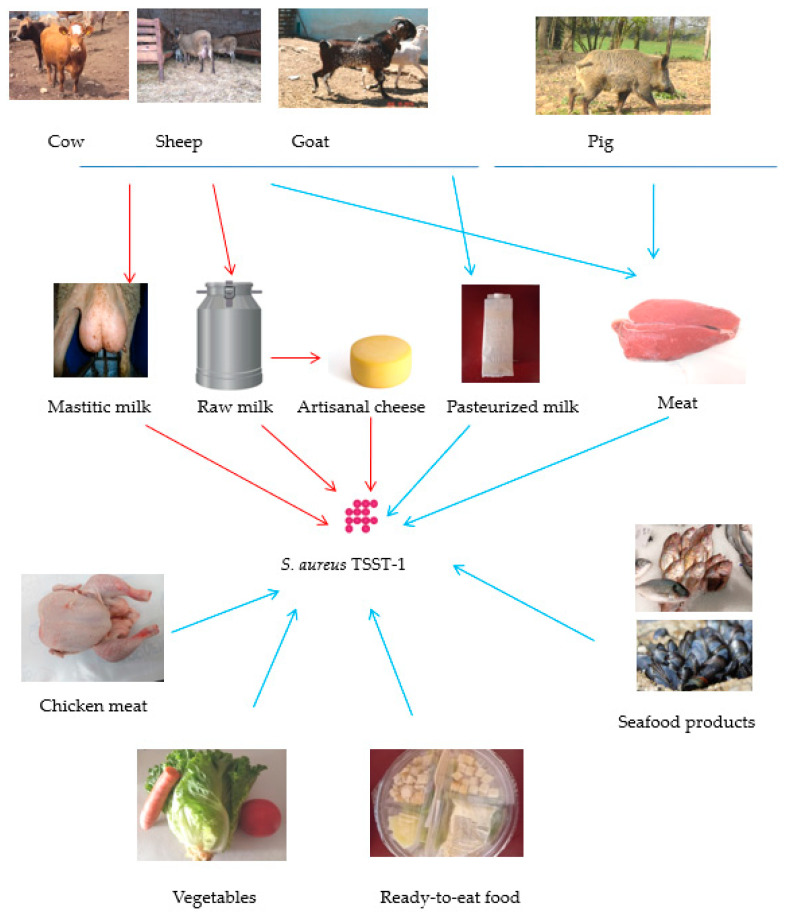
Occurrence of *S. aureus* TSST-1 in foods. High and low occurrence of *S. aureus* TSST-1 are indicated with red and blue arrows, respectively.

The occurrence of *S. aureus* TSST-1 in foods is presented in Table 3. *S. aureus* TSST-1 strains have been identified in various foods, indicating that foods can serve as vectors for the dissemination of this pathogen to consumers. The pathogen is predominantly found in foods of animal origin. According to published studies conducted in several countries, the majority of foods contaminated with *S. aureus* TSST-1 are dairy products, in particular raw milk and cheese produced from raw milk (artisanal cheeses) (Figure 2). The lowest incidence of *S. aureus* TSST-1 has been reported in meat and fish.

Comparable data from published review studies regarding the occurrence of *S. aureus* TSST-1 in foods were not found. In contrast, reviews on the presence of *S. aureus* in foods have been published in various countries. According to a review made by Liang et al. [98] with meta-analysis of the presence of *S. aureus* in various foods based on published studies between the years 2012 and 2022, the highest prevalence of the pathogen was recorded in cereals (46.3%), followed by meat (35.7%). The same authors also reported that the prevalence of *S. aureus* in dairy and seafood products was 28.4% and 24.8%, respectively. According to another review completed with meta-analysis of data from studies published between 2012 and 2022 [99], *S. aureus* was mostly prevalent in ready-to-eat food (35.1%), followed by meat (21.7%), and dairy products (18.5%). Based on meta-analysis data from 24 studies conducted in different countries between the years 1980 and 2021, the prevalence of *S. aureus* in raw milk samples was 11.6% [4]. The differences in prevalence of *S. aureus* in foods found in the meta-analysis of published studies may be due to factors such as the different number of analyzed studies, the study periods, the variety of analyzed foods, different analytical methods used, and the overall quality of published data [99].

Although Liang et al. [98] reported a high prevalence of *S. aureus* TSST-1 in cereals (46.3%), Wang et al. [6] did not detect the *tst* gene in any of 24 S. aureus isolates obtained from 224 examined samples of infant rice cereals in China. They also reported that all 54 *S. aureus* isolates from infant formula milk and infant rice cereal were positive (n = 34, 62.96%) for the classical SE genes but negative for the *tst* gene, concluding that these enterotoxigenic *S. aureus* strains may be a potential risk to infant health. Therefore, the occurrence of *S. aureus* harboring the *tst* gene in foods may differ from the presence of *S. aureus* in foods, due to reasons such as differences in the acquisition of toxigenic SE genes in the pathogen strains found in foods [17].

### 4.1. Milk

Several studies accomplished in various countries worldwide have indicated the occurrence of *S. aureus* TSST-1 in bovine, ovine, and caprine raw milk. In agreement with the present study, milk has been considered as a primary source of human exposure to *S. aureus* and its enterotoxins [4,100,101]. The majority of studies on the presence of the *S. aureus* TSST-1 have reported a higher occurrence in mastitic milk than in raw milk or pasteurized milk. The pathogen’s ability to infect the udder is due to factors such as toxin production, invading enzymes of udder skin, or ability to form biofilm on udder skin [43,102]. Among superantigens, TSST-1 enhances mammary inflammation via excessive cytokine production, resulting in mastitis [27,57]. Rainard et al. [103] reported that *S. aureus* enzymes and hemolysins are involved in injuring the udder epithelium, and the resistance to phagocytosis constitutes an important element of *S. aureus* pathogenicity in mastitis. *S. aureus* Hla, Hlb, and δ toxins damage cell membranes, platelets, and lysosomes and cause ischemia and necrosis [5]. *S. aureus* enzymes coagulase and hyaluronidase are involved in abscess formation and udder skin penetration, while *S. aureus* lipases degrade skin lipids [104]. Among leucocidins of *S. aureus*, the PVL is the toxin with the highest ability to inactivate immune cells [18]. Aguirre-Sánchez et al. [55] reported that the mastitis biofilm cassette *icaABCD* played an important role in the adhesion of *S. aureus* to the cow mammary epithelium. Other factors enabling mammary gland infection are also improper milking equipment, poor hygiene milking conditions, teat lesions, or antibiotic resistance of infecting *S. aureus* strains [31,105]. Thus, mastitic milk from *S. aureus* TSST-1 clinically or sub-clinically infected animals can contaminate the farm bulk milk and potentially spread the pathogen to consumers.

In contrast to the high occurrence of *S. aureus* TSST-1 in raw milk, very few research articles have reported the presence of the pathogen in pasteurized milk. Among 258 samples of pasteurized milk obtained in different cities in China, *S. aureus* was isolated in 12 of the pasteurized milk samples, while *S. aureus* TSST-1 was identified in only 1 sample [74]. Chaalal et al. [75] examined pasteurized milk sold in the market of Algeria during 2014–2015 and detected *S. aureus* TSST-1 in 1 (7.1%) isolate out of 14 *S. aureus* isolates. Zhang et al. [106] reported that among 140 published studies (1992–2021) conducted in various countries, the prevalence of *S. aureus* in raw milk (33.36%) was higher than that in pasteurized milk (7.66%).

*S. aureus* can contaminate the raw milk either by direct excretion from ruminants’ mammary glands with clinical or subclinical staphylococcal mastitis or via environmental contamination due to improper handling or inappropriate heat processing of raw milk. Raw milk is a good substrate for *S. aureus* growth due to nutritional components such as proteins or fatty acids that enhance the survival of the pathogen [96,107]. *S. aureus* cells are inactivated during the milk pasteurization process, and the pathogen is usually found in pasteurized milk as a result of post-pasteurization contamination [108,109]. Therefore, low population levels of *S. aureus* have usually been found in pasteurized milk [106]. Additionally, *S. aureus* levels in pasteurized milk are also influenced by storage temperature, a failure of the pasteurization process, or post-pasteurization contamination [104]. Non-thermal sterilization processes, such as electric fields, are also used for the elimination of pathogens including *S. aureus* in milk [110,111,112]. The reutilization of waste milk may also be a risk for the *S. aureus* presence in milk [113].

### 4.2. Cheese

The occurrence of *S. aureus* TSST-1 in raw milk or artisanal cheeses, in contrast to pasteurized milk cheeses, has been confirmed in several studies. (Table 3). Raw milk and artisanal cheeses are produced in many countries due to their distinct aroma and flavor. Pasteurization can destroy certain milk components (e.g., heat-sensitive enzymes, aldehydes, or vitamins) as well as the natural microbiota of milk [114]. However, the microbial safety of raw milk and artisanal cheeses is critical since pathogens such as *S. aureus* are not inactivated by pasteurization and may survive during cheese ripening [115]. The starter culture can affect the growth of *S. aureus* TSST-1 during cheese ripening. Thus, a starter culture of lactic acid bacteria (LAB), *Weissella paramesenteroides* or *Lactobacillus rhamnosus*, reduced *tst* and *sec* expression in experimental cheeses from day one to day seven (*p* < 0.05) during ripening [116]. Since *S. aureus* cannot survive during milk pasteurization, the occurrence in cheeses made from pasteurized milk may be due to poor hygienic practices in the dairy plant or from cross-contamination as the pathogen is also part of the human microflora [106]. However, in 54 *S. aureus* isolates from artisanal Coalho Cheese made from goat milk in Brasil, the *tst* gene was not detected [78]. Zhang et al. [117] also reported that the prevalence rate of *S. aureus* in dairy products was high in South America (49.03%) and low in North America (<3.00%), while prevalence rates of 19.11%, 9.60%, 7.01% were found for Asia, Africa, and Europe, respectively, and concluded that proper pasteurization of milk along with good hygienic practices in dairy plants should be implemented, and pathogen surveillance in dairy products is also required.

### 4.3. Meat

According to several studies, the presence of *S. aureus* was found in the tonsils, pharynx, mouth, udder skin, nasal cavity, rectum, or vaginal mucous membranes of sheep, goats, cows, or pigs [2,118,119,120,121,122]. *S. aureus* is also a commensal bacterium of chicken microflora [123,124]. *S. aureus* presence was also verified in beef, sheep, goat, pork, chicken, or turkey meat [125,126,127]. Contamination of meat with *S. aureus* may be due to infected animals entering slaughterhouses, poor hygienic slaughtering practices, contamination from *S. aureus* carriers among workers, or exposure to contaminated environmental sources during slaughter and improper hygienic handling or processing of the meat [122]. Léguillier et al. [101] conducted a meta-analysis of published studies between 2012 and 2022 and reported that the prevalence of *S. aureus* in meat products was 59.51%, and among the contaminated meat products, the prevalence was found in beef (44%), pork meat (28%), chicken (22%), and turkey (6%). After a meta-analysis of published studies between 2000 and 2016, the pooled prevalence rates of *S. aureus* MRSA and *S. aureus* presence in raw meats overall were 3.2% and 29.2%, respectively [102]. The pooled prevalence rates of *S. aureus* presence in chicken, beef, and pork meat were 34.5%, 29.7%, and 23.5%, respectively [102]. After *tst* gene analysis, no *S. aureus* TSST1 strains were identified among 289 *S. aureus* isolates in chicken meat (China), among 76 *S. aureus* isolates in beef meat (USA), nor among 15 and 7 *S. aureus* isolates in beef burgers and hot dogs (Egypt), as reported by Li et al. [128], Abdalrahman et al. [83], and Mahros et al. [84], respectively. According to the present review, *S. aureus* TSST-1 occurrence records ranging from 9.52% up to 100% were found in beef, pork, and lamb meat in different countries (Table 3).

### 4.4. Fish

Arfatahery et al. [87] examined 300 fish samples sold in the market of Iran and isolated *S. aureus* in 122 (40.7%) samples. Among the fish *S. aureus* isolates, eight (3.9%) isolates were characterized as *S. aureus* TSST-1. Puah et al. [37] found the *tst* gene in four (12.5%) and one (5%) isolates out of two *S. aureus* isolates from sushi and sashimi fish samples, respectively. Polluted water with *S. aureus* and improper hygienic status during fish capture, handling, processing, and transportation may be reasons for fish contamination with the pathogen [129].

### 4.5. tst Gene

The *tst* gene in *S. aureus* was found combined with other virulence genes, resistance genes to antibiotics, or adhesion-biofilm formation genes, either in single form or in combined gene groups [64,130]. Among 291 *S. aureus* isolates from milk and Minas cheese, the *tst* gene was identified in 8 (2.5%) isolates, the classical *seb* gene was found in 11 (3.8%) isolates, while the non-classical *seg* and *sei* genes were detected in 51 (17.5%) and 40 (13.7%) isolates, respectively [49]. Puah et al. [37] analyzed 52 *S. aureus* isolates from Sushi and Sashimi fish samples and reported the following gene occurrence: *tst* 5 (9.6%), *hla* 47 (90.4%), *hld* 31 (59.6%), *lukDE* 20 (38.5%), 21 *hlgv* (30.8%), and *lukPV* 5 (9.6%). Similarly, Yang et al. [131] isolated 69 *S. aureus* in retail food in China and detected *pvl*, *eta*, *etb*, and *tst* genes in eight (11.6%), seven (10.1%), seven (10.1%), and five (7.2%) isolates, respectively. Mokhtari et al. [38] found that in 36 *S. aureus* of mastitic ovine milk, the *mecA* and *tst* genes were present in 10 (27.77%) and 5 (13.88%) isolates, respectively.

Mehli et al. [80] reported that among 40 *S. aureus* isolates from dairy products, the *tst* gene was found in combination with other toxigenic genes: *sea*, *sec*, *tst* from raw milk (n = 1); *seb*, *sec*, *tst* from whey (n = 1); *sec*, *seg*, *tst* from whey cheese (n = 2); *sec*, *seh*, *tst* from cheese (n = 1); *seg*, *seh*, *tst* from raw milk (n = 1); and *sec*, *seg*, *seh*, *tst* from raw milk (n = 3). Costa et al. [40] reported that among 38 *S. aureus* isolates from cow mastitic milk, 25 (65.78%) isolates carried combined toxin genes of *seb*, *sec*, *sed*, *tst*, and the *icaD* adhesion gene. Among three *S. aureus* isolates obtained from food samples (cheese, eggs, and infant food) in Mexico the *tst* gene was combined with other genes in one (33.33%) isolate with *saK*, *seA*, *seE*, one *tst*; one (33.33%) isolate with *saK* and *tst;* and one (33.33%) isolate with *saK*, *seA*, and *tst* genes [97]. Among 78 *S. aureus* isolates from mastitic cow milk, 8 isolates (10.3%) were positive for *sed*, *sei*, *sem*, and *sen*; 6 (7.7%) isolates possessed *sed*, *seg*, *sei*, *sem*, *sen*, and *tst*; 5 (6.4%) isolates had *sei*, *sem*, and *sen*; and 4 (5.1%) isolates had *sei* and *sen* [48]. The combined *tst* gene profile of 46 *S. aureus* isolates from meat (beef, pork, lamb) from slaughtered animals in Iraq revealed a group gene profile of *seA*, *seB*, *seC*, and *tst* (6, 11.3%) and a group gene profile of *seA*, *seB*, and *tst* (10/21.7%) [85]. Among eight MRSA isolates obtained from retail raw meat in Japan, seven of them were detected with combined presence of *sec*, *sel*, and *tst* genes [81]. Ogata et al. [81] also reported that two MRSA isolates from raw meat in Japan were found with a combined presence of *tst*, *sec*, and *sel*, which encode superantigen toxins. A combined group of *seo*, *tst*, and *etB* genes in 10 (28.0%) isolates from 35 *S. aureus* were found in mastitic cow milk in China [16]. Among 15 *S. aureus* PVL isolates from mastitic bovine milk in Turkey, 2 (13.3%) isolates were identified with *etd*, *luk-PV*, *tst-1*, and *eta* genes [51].

The occurrence of *S. aureus* TSST-1 in various foods poses a significant risk for contamination and subsequent transmission of the pathogen to consumers. Since the *tst* gene is often found in combination with other toxigenic genes, antibiotic resistance genes, or biofilm formation genes, the risk of disseminating the pathogen to humans is also high. *S. aureus* TSST-1 can transfer the *tst* gene to other bacteria through transductions by means of bacteriophages [27]. MRSA is a leading antimicrobial-resistant pathogen responsible for deaths in hospitals worldwide [39]. The infected patients from the TSST-1-producing MRSA are difficult to treat and relative infections may also become fatal [53]. The common presence of PVL and TSST toxins has been associated with unfavorable treatment outcomes of *S. aureus* infections [132]. Since toxigenic genes are usually found in the *S. aureus* microbiome with antibiotic resistance genes, the pathogen infection treatment is generally a difficult process [39].

The growth of *S. aureus* in foods is inhibited due to factors such as antagonistic activity of other microorganisms, including LAB, refrigerated storage temperatures, low pH (usually below 4.6), or nitrate presence, e.g., in cured meat. *S. aureus* cells are usually eliminated during the pasteurization process (pasteurization of milk at 71.6 °C for 16 s), although most of the enterotoxins, including TSST-1, are stable during a pasteurization or even sterilization temperature process [133,134]. He et al. [135] reported that oxidative inactivation of bacteria by using plasma-activated water is an alternative method for the elimination of pathogens in foods with high biosafety. The production of most *S. aureus* toxins, including TSST-1, is achieved after pathogen growth at levels of 5–6 log CFU/g foods [136]. According also to European Union legislation, the various types of dairy products (cheeses, milk, and whey powder) must be examined for the presence of SEs if coagulase-positive *Staphylococcus* populations are higher than 5 log CFU/g (Regulation EC, No 2073/2005) [137]. If SEs are detected in a food sample (25 g), the food is considered unsafe for consumption and must be withdrawn from the market.

### 4.6. TSST-1 in Foods

According to our literature review, no published study with the presence of formed TSST-1 in foods was found, except for the occurrence of *S. aureus* TSST-1 cells. However, *S. aureus* TSST-1 outbreaks due to consumption of contaminated foods were reported in various countries. In 2009, a case of *S. aureus* TSST-1 food outbreak was registered in Sicily, Italy [62]. The TSST-1 symptoms were observed in two patients following the consumption of the ovine Primosale cheese. Molecular analysis of *S. aureus* isolate revealed the presence of *sec* and *tst* genes. Chiang et al. [138] examined 147 *S. aureus* strains isolated from patients involved in SFP outbreaks that occurred during 2001–2003 in Taiwan. PCR analysis of the 147 *S. aureus* isolates revealed that the predominant gene was *tst* (87, 59.1%), while the rest of the identified genes were *sea* (43, 29.2%), *seb* (29, 19.7%), *sec* (10, 6.8%), and *sed* (3.2.0%). In 2021, a 15-year-old boy was admitted to a hospital with fever, confusion, and TSST-1 symptoms that started one day after consumption of meat in a fast-food restaurant in Belgium [139]. The *S. aureus* (t2509) was identified as the causative agent, which was previously associated with a foodborne *S. aureus* outbreak in St Petersburg in 2013. Cha et al. [140] reported a 12% *S. aureus* TSST-1 prevalence in *S. aureus* isolates obtained from stool samples of patients affected by foodborne illness in Korea.

Since TSST-1 is usually stable in foods, detoxification strategies are challenging and focus on prevention rather than post-toxin elimination. Prevention strategies should involve the application of hygienic control measures to avoid the presence of *S. aureus* TSST-1 in foods.

## 5. Conclusions

*S. aureus* TSST-1 has been found in various foods, and TSS is a severe disease caused by TSST-1 and associated with SFP. Control measures should be applied to avoid dissemination of the pathogen to consumers. Control of mastitis in lactating animals is crucial to avoid contamination of raw milk with the pathogen. In order to assess risks and contamination routes of *S. aureus* TSST-1 in foods, further studies on the occurrence of the pathogen in food products are required. Since the *tst* gene is often found combined with other toxigenic genes, antibiotic resistance genes, or biofilm formation genes the protection of consumers from multi-drug-resistant *S. aureus* TSST-1 strains is an important and crucial issue for public health. Moreover, genetic studies on food *S. aureus* TSST-1 strains, in relation to clinical isolates, require comprehensive genomic and epidemiological studies. Harmonized global surveillance of *S. aureus* TSST-1 in foods is required to combat consumer contamination. Since TSST-1 is found in low levels in foods, sensitive analytical methods should be further improved. Public health policy related to *S. aureus* TSST-1 should involve surveillance, prevention strategies, and outbreak reporting. Key pillars for future research on S. aureus TSST-1 presence in foods should include improving diagnostic methods, preventing mastitis in animals and contamination of raw milk, evaluating food safety risks associated with the pathogen, and developing effective preventive methods for contamination of foods with the pathogen.

## Figures and Tables

**Table 1:** *S. aureus* toxins’ activity.

Toxins	Activity
Staphylococcal enterotoxins (SEs)	Superantigen with emetic activity
SE-like toxins	Superantigen and no emetic activity
Hemolysins (Hla and Hlb)	Pore-forming
Leukocidins (PVL, LukAB, LukED, LukPQ and LukMF)	Damage of immune cells and pore-forming
Exfoliative toxins (ETA-ETE)	Skin exfoliation and blistering by cleaving desmoglein 1

**Table 2:** Isolation and identification methods for S. aureus TSST-1 in foods.

Method Category	Method	Target	Description	References
Culture-based methods	Baird–Parker agar with egg yolk tellurite	*S. aureus*	Selective isolation based on colony morphology	[28,29,30]
	Mannitol Salt Agar (MSA)	*S. aureus*	Selective and differential growth via mannitol fermentation	[28,29]
	Columbia blood agar	*S. aureus*	Evaluation of hemolytic activity	[31]
Phenotypic and biochemical methods	Gram staining	*Staphylococci*	Morphological and Gram reaction characterization	[31]
	Catalase test	*Staphylococci*	Detection of catalase activity	[31]
	API ID 32 Staph	*S. aureus*	Biochemical identification system	[31]
Molecular methods	Conventional PCR (*tst* gene)	TSST-1	Detection of the *tst* gene	[27,35,37,38]
	Multiplex PCR	TSST-1, SEs, *mecA*	Simultaneous detection of virulence and resistance genes	[39,40]
	Real-time PCR	TSST-1	Rapid and quantitative *tst* gene detection	[28]
	Commercial kits (e.g., QIAamp DNA Mini Kit)	*S. aureus* DNA	Silica membrane-based DNA extraction	[36]
	Phenol-chloroform extraction	*S. aureus* DNA	Organic solvent-based DNA purification	[36]
Immunological methods	Reversed passive latex agglutination (TST-RPLA-TD940)	TSST-1	Antigen–antibody agglutination assay	[27]
	ELISA (RIDASCREEN^®^, VIDAS^®^ SET2)	Classical SEs	Immuno-enzymatic detection of enterotoxins	[28]
Biosensor-based methods	Polymer-based biosensors	*S. aureus*, TSST-1	Rapid biosensing platforms	[32]
	Aptamer-based biosensors	*S. aureus*	Specific oligonucleotide-target interaction	[33]
Isothermal amplification	RPA–lateral flow assay	*S. aureus*	Rapid isothermal amplification with visual readout	[34]
Mass spectrometry	MALDI-TOF MS	*S. aureus*	Protein fingerprint-based identification	[41]
Advanced molecular (genomic) methods	Whole-Genome Sequencing	TSST-1, virulence genes	Genome-level characterization	[27,42]
Bioassay methods	T-cell activation bioassay	TSST-1	Functional assessment of superantigen activity	[27,43]
Analytical chemistry methods	LC–HRMS	Staphylococcal enterotoxins	High-resolution toxin detection	[35,44]

**Table 3:** Occurrence of *S. aureus* TSST-1 in foods.

Product/Sample	Year	Country	*S. aureus* (Ν)	*S. aureus* TSST-1 (n, %)	References
Mastitic cow milk					
	2000	Germany	103	21 (20.38%)	[45]
	2000–2001	Japan	270	183 (67.8%)	[46]
	2001	Norway	101	24 (23.8%)	[47]
	2003–2004	USA	78	20 (25.6%)	[48]
	2010	Brazil	125	0	[49]
	2011	USA	83	7 (8.4%)	[50]
	2013	China	35	10 (28%)	[16]
	2013	Turkey	15	2 (13.3%)	[51]
	2015	Poland	124	3 (2.4%)	[52]
	2017	Brazil	38	25 (65.78%)	[40]
	2017–2022	South Korea	30 (MRSA)	4 (13.33%)	[53]
	2018	Brazil	285	105 (36.8%)	[54]
	2024	Mexico	50	26 (52%)	[55]
	2024	Ethiopia	63	7 (10.9%)	[56]
Mastitic sheep milk					
	1991	Spain	108	80 (74%)	[57]
	2017	Iran	36	5 (13.88%)	[38]
	2018	Iran	58	16 (27.59%)	[58]
Raw cow milk					
	1996	Japan	35	10 (28.6%)	[59]
	2001	Norway	101	24 (23.8%)	[47]
	2010	Brazil	96	6 (6.2%)	[49]
	2010	Ireland	37	11 (29.72%)	[60]
	2012	Italy	153	0	[61]
	2013–2014	Tunisia	27	4 (14.8%)	[13]
	2013	China	35	10 (28%)	[16]
	2015	Ethiopia	100	10 (10%)	[36]
	2015	Italy	25	9 (36%)	[62]
	2021–2022	Iran	70	2 (2.56%)	[63]
	2022	Portugal	22	1 (4.8%)	[64]
	2024	Poland	6	1 (8%)	[65]
	2018	Czech Republic	11	1 (9.09%)	[66]
Raw sheep milk					
	2003	Switzerland	31	6 (19.35%)	[67]
	2004–2007	Slovakia	49	6 (12.2%)	[68]
	2010	Ireland	1	0	[60]
	2014	Greece	34	1 (2.94%)	[7]
	2015	Italy	42	13 (30.95%)	[62]
Raw goat milk					
	1986	France	52	1 (1.92%)	[69]
	1991	Spain	64	40 (62.5%)	[70]
	2001	Norway	95	52 (54.7%)	[47]
	2003	Switzerland	262	107 (40.95%)	[67]
	2010	Ireland	10	2 (20%)	[60]
	2014	Greece	11	1 (9.09%)	[7]
	2015	Italy	19	8 (42.10%)	[62]
	2016–2017	China	68	17 (36.76%)	[71]
	2022	China	13, 6, 4, 3	3 (23.1%)	[72]
Pasteurized milk					
	2003–2004	Germany	9 (goat cheese)	4 (47.36%)	[73]
	2011–2016	China	258/12	1 (8.3%)	[74]
	2014–2015	Algeria	14	1 (7.1%)	[75]
Raw buffalo milk					
	2011	Brazil	6	5 (83.33%)	[50]
	2022	China	6	0	[72]
Other raw milks	2022	China	4 (camel), 3 (yak)	0, 0	[72]
Human milk	2021	Brazil	15	1 (6.7%)	[76]
Cheeses					
Raw sheep cheese	2004–2007	Slovakia	24	10 (41.7%)	[68]
Bryndza cheese	2004–2007	Slovakia	6	2 (33.3%)	[68]
Sheep cheese	2012	Italy	100	29 (29%)	[77]
Minas frescal cheese	2010	Brazil	70	2 (2.9%)	[49]
Goat artisanal coalho cheese	2017	Brazil	54	0	[78]
Artisan cheese	2018–2019	Belgium	24	9 (37.5%)	[79]
Raw milk cheese	2024	Poland	13	4 (34.7%)	[65]
Sheep cheese	2015	Italy	4	4 (100%)	[62]
Cow cheese	2015	Italy	1	1 (100%)	[62]
Ricotta cheese	2015	Italy	0	0	[62]
Local cheese	2018	Iran	22	2 (9.09%)	[23]
Whey cheese	2016	Norway	22	2 (9.09%)	[80]
Meat and meat products					
Meat (raw)	2008–2009	Japan	8	7 (88%)	[81]
Meat (raw)	2003–2009	Japan	7	2 (28.57%)	[81]
Meat (raw)	2014	Tunisia	2	1 (50%)	[82]
Beef meat	2014	USA	76	0	[83]
Beef livers	2014	USA	143	4 (2.8%)	[83]
Pork meat	2014	USA	115	15 (13%)	[83]
Beef burger	2020	Egypt	15	0	[84]
Hot dog	2020	Egypt	7	0	[84]
Minced meat	2015	Italy	3	1 (33.33%)	[62]
Bovine meat	2015	Italy	2	2 (100%)	[62]
Meat products	2009–2011	Ivory Coast	42	16 (38.2%)	[41]
Beef, pork, lamb	2024	Iraq	46	29 (63%)	[85]
Pig tongues	2019	China	21	2 (9.52%)	[86]
Fish and seafoods					
Sushi	2014	Malaysia	32	4 (12.5%)	[37]
Sashimi	2014	Malaysia	20	1 (5%)	[37]
Fishery products (shrimp, fish)	2013–2014	Japan	122	3 (8.3%)	[87]
Freshwater fish	2015–2016	China	74	3 (4.05%)	[88]
Shrimp	2022	China	63	30 (47.6%)	[89]
Oysters	2024	Egypt	33	5 (16.7%)	[90]
Ready-to-eat raw fish	2011	Japan	175	25 (14.2%)	[91]
Ready-to-eat foods					
Various foods	2006	Korea	285	38 (13.5%)	[92]
Ready-to-eat foods	2018–2019	Algeria	48	1 (2.1%)	[93]
Ready-to-eat pizza	2022–2023	Egypt	152	0	[94]
Various foods (raw milk, egg, chicken, fish, RTE)	2022	Bangladesh	100	20 (20%)	[95]
Food products (dairy, meat, other)	2006–2007	Iran	100	12 (12%)	[96]
Food preparations	2015	Italy	6	5 (83.33%)	[62]
Various foods (meat, fish, confectionery, ham)	2013	Czech Republic	93	28 (30%)	[97]
Foods (cheese, eggshell, infant food)	2019	Mexico	17	3 (17.64%)	[98]
Various food products	2014–2015	Algeria	153	5 (3.2%)	[75]

N = total number of *S. aureus* isolates; n = number of *S. aureus* TSST-1 isolates.

## Data Availability

No new data were created or analyzed in this study. Data sharing is not applicable to this article.

## References

[1] Touaitia R., Mairi A., Ibrahim N.A., Basher N.S., Idres T., Touati A. (2025). *Staphylococcus aureus*: A Review of the Pathogenesis and Virulence Mechanisms. Antibiotics.

[2] European Food Safety Authority (2021). The European Union one Health 2020 Zoonoses Report. EFSA J..

[3] Li Q., Dou L., Zhang Y., Luo L., Yang H., Wen K., Yu X., Shen J., Wang Z. (2024). A comprehensive review on the detection of *Staphylococcus aureus* enterotoxins in food samples. Compr. Rev. Food Sci. Food Saf..

[4] Shalaby M., Reboud J., Forde T., Zadoks R.N., Busin V. (2024). Distribution and prevalence of enterotoxigenic *Staphylococcus aureus* and staphylococcal enterotoxins in raw ruminants’ milk: A systematic review. Food Microbiol..

[5] Abril A.G., Villa T.G., Barros-Velázquez J., Cañas B., Sánchez-Pérez A., Calo-Mata P., Carrera M. (2020). *Staphylococcus aureus* exotoxins and their detection in the dairy industry and mastitis. Toxins.

[6] Wang X., Meng J., Zhang J., Zhou T., Zhang Y., Yang B., Xi M., Xia X. (2012). Characterization of *Staphylococcus aureus* isolated from powdered infant formula milk and infant rice cereal in China. Int. J. Food Microbiol..

[7] Pexara A., Solomakos N., Sergelidis D., Angelidis A., Govaris A. (2016). Occurrence and antibiotic resistance of enterotoxigenic *Staphylococcus aureus* in raw ovine and caprine milk in Greece. Dairy Sci. Technol..

[8] Lim K.L., Khor W.C., Ong K.H., Timothy L., Aung K.T. (2023). Occurrence and patterns of enterotoxin genes, spa types and antimicrobial resistance patterns in *Staphylococcus aureus* in food and food contact surfaces in Singapore. Microorganisms.

[9] Di Bella S., Marini B., Stroffolini G., Geremia N., Giacobbe D.R., Campanile F., Michele Bartoletti M., Alloisio G., di Risio L., Viglietti G. (2025). The virulence toolkit of *Staphylococcus aureus*: A comprehensive review of toxin diversity, molecular mechanisms, and clinical implications. Eur. J. Clin. Microbiol. Infect. Dis..

[10] Zheng Y., Qin C., Zhang X., Zhu Y., Li A., Wang M., Tang Y., Kreiswirth B.N., Chen L., Zhang H. (2020). The *tst* gene associated *Staphylococcus aureus* pathogenicity island facilitates its pathogenesis by promoting the secretion of inflammatory cytokines and inducing immune suppression. Microb. Pathog..

[11] Strom M.A., Hsu D.Y., Silverberg J.I. (2017). Prevalence, comorbidities and mortality of toxic shock syndrome in children and adults in the USA. Microbiol. Immunol..

[12] Sharma H., Smith D., Turner C.E., Game L., Pichon B., Hope R., Hill R., Kearns A., Sriskandan S. (2018). Clinical and Molecular Epidemiology of Staphylococcal Toxic Shock Syndrome in the United Kingdom. Emerg. Infect. Dis..

[13] Khemiri M., Abbassi M.S., Couto N., Mansouri R., Hammami S., Pomba C. (2018). Genetic characterization of *Staphylococcus aureus* isolated from milk and nasal samples of healthy cows in Tunisia: First report of ST97-t267-agrI-SCCmecV MRSA of bovine origin in Tunisia. J. Glob. Antimicrob. Resist..

[14] Parrish K.L., Wylie K.M., Reich P.J., Hogan P.G., Wylie T.N., Kennedy C.R., Lainhart W., Hunstad A.D., Burnham C.-A.D., Fritz A.S. (2019). Carriage of the toxic shock syndrome toxin gene by contemporary community-associated *Staphylococcus aureus* isolates. J. Pediatr. Infect. Dis. Soc..

[15] Denayer S., Delbrassinne L., Nia Y., Botteldoorn N. (2017). Food-borne outbreak investigation and molecular typing: High diversity of *Staphylococcus aureus* strains and importance of toxin detection. Toxins.

[16] Wang D., Zhang L., Yong C., Shen M., Ali T., Shahid M., Han K., Zhou X., Han B. (2017). Relationships among superantigen toxin gene profiles, genotypes, and pathogenic characteristics of *Staphylococcus aureus* isolates from bovine mastitis. J. Dairy Sci..

[17] Aung M.S., Urushibara N., Kawaguchiya M., Ito M., Habadera S., Kobayashi N. (2020). Prevalence and genetic diversity of Staphylococcal enterotoxin (-like) genes *sey*, *selw*, *selx*, *selz*, *sel26* and *sel27* in community-acquired Methicillin-Resistant *Staphylococcus aureus*. Toxins.

[18] Badiou C., Dumitrescu O., George N., Forbes A.R.N., Drougka E., Chan K.S., Ramdani-Bouguessa N., Meugnier H., Bes M., Vandenesch F. (2010). Rapid detection of *Staphylococcus aureus* Panton-Valentine Leucocidin in clinical specimens by enzyme-linked immunosorbent assay and immunochromatographic tests. J. Clin. Microbiol..

[19] Wang F., Hongjun Y., Hong-Bin H., Changfa W., Yundong G., Qifeng Z., Xiaohong W., Yanjun Z. (2011). Study on the hemolysin phenotype and the genotype distribution of *Staphylococcus aureus* caused bovine mastitis in Shandong dairy farms. Int. J. Appl. Res. Vet. Med..

[20] Oliveira D., Borges A., Simões M. (2018). *Staphylococcus aureus* toxins and their molecular activity in infectious diseases. Toxins.

[21] Bokarewa M.I., Jin T., Tarkowski A. (2006). *Staphylococcus aureus*: Staphylokinase. Int. J. Biochem. Cell Biol..

[22] Idrees M., Sawant S., Karodia N., Rahman A. (2021). Staphylococcus aureus biofilm: Morphology, genetics, pathogenesis and treatment strategies. Int. J. Environ. Res. Public Health.

[23] Ghasemi P., Mahdavi S. (2018). Study of prevalence of Toxic Shock Syndrome Toxin (TSST-1) and Methicilin Resistance (MecA) genes of *Staphylococcus aureus* isolates from local Cheese in northwest of Iran. Gen. Cel. Tissue.

[24] Andrey D.O., Jousselin A., Villanueva M., Renzoni A., Monod A., Barras C., Rodriguez N., Kelley W.L. (2015). Impact of the Regulators *SigB*, *Rot*, *SarA* and *sarS* on the Toxic Shock Tst Promoter and TSST-1 Expression in *Staphylococcus aureus*. PLoS ONE.

[25] Chiaruzzi M., Barbry A., Muggeo A., Tristan A., Jacquemond I., Badiou C., Cluzeau L., Bourdeau S., Durand T., Engelmann A. (2020). Vaginal tampon colonization by *Staphylococcus aureus* in healthy women. Appl. Environ. Microbiol..

[26] Atchade E., de Tymowski C., Grall N., Tanaka S., Montravers P. (2024). Toxic Shock Syndrome: A literature review. Antibiotics.

[27] Touaitia R., Ibrahim N.A., Touati A., Idres T. (2025). *Staphylococcus aureus* in bovine mastitis: A narrative review of prevalence, antimicrobial resistance, and advances in detection strategies. Antibiotics.

[28] Mairi A., Ibrahim N.A., Idres T., Touati A. (2025). A Comprehensive review of detection methods for *Staphylococcus aureus* and its Enterotoxins in Food: From traditional to emerging technologies. Toxins.

[29] Tallent S.M., Bennett R.W., Miller J.M. (2022). Bacteriological Analytical Manual Chapter 13B: Staphylococcal Enterotoxins Detection Methods (September 2022 Edition).

[30] (2021). Microbiology of the food chain—Horizontal method for the enumeration of coagulase-positive staphylococci (Staphylococcus aureus and other species)—Part 1: Method using Baird-Parker agar medium.

[31] Campos B., Pickering A.C., Rocha L.S., Aguilar A.P., Fabres-Klein M.H., de Oliveira Mendes T.A., Fitzgerald J.R., de Oliveira Barros Ribon A. (2022). Diversity and pathogenesis of *Staphylococcus aureus* from bovine mastitis: Current understanding and future perspectives. BMC Vet. Res..

[32] Gao Y., Zhang S., Aili T., Yang J., Jia Z., Wang J., Li H., Bai L., Lv X., Huang X. (2022). Dual signal light detection of beta-lactoglobulin based on a porous silicon bragg mirror. Biosens. Bioelectron..

[33] Gan Z., Roslan M.A.M., Shukor M.Y.A., Halim M., Yasid N.A., Abdullah J., Yasin I.S.M., Wasoh H. (2022). Advances in aptamer-based biosensors and cell-internalizing SELEX technology for diagnostic and therapeutic application. Biosensors.

[34] Zhang Y., Liu X., Luo J., Liu H., Li Y., Liu J., Zhu L., Wang J., Zeng H. (2025). Dual recombinase polymerase amplification system combined with lateral flow immunoassay for simultaneous detection of *Staphylococcus aureus* and *Vibrio parahaemolyticus*. J. Pharm. Biomed. Anal..

[35] Cieza M.Y.R., Bonsaglia E.C.R., Rall V.L.M., Santos M.V.d., Silva N.C.C. (2024). Staphylococcal enterotoxins: Description and importance in food. Pathogens.

[36] Tarekgne E.K., Skjerdal T., Skeie S., Rudi K., Porcellato D., Félix B., Narvhus J.A. (2016). Enterotoxin gene profile and molecular characterization of *Staphylococcus aureus* isolates from bovine bulk milk and milk products of Tigray region, northern Ethiopia. J. Food Prot..

[37] Puah S.M., Chua K.H., Tan J.A.M.A. (2016). Virulence factors and antibiotic susceptibility of *Staphylococcus aureus* isolates in ready-to-eat foods: Detection of *S. aureus* contamination and a high prevalence of virulence genes. Int. J. Environ. Res. Public Health.

[38] Mokhtari A., Ebrahim Kahrizangi A., Hasani P. (2018). Genomic identification of Toxic shock syndrome producing and methicillin resistant *Staphylococcus aureus* strains in human and sheep isolates. J. Hellenic Vet. Med. Soc..

[39] Crippa B.L., Camargo C.H., Mores Rall V.L.M., Silva N.C.C. (2025). MRSA: The relationship between animal source foods (ASF) and humans–A One Health Concern. Curr. Food Sci. Tech. Rep..

[40] Costa F.N., Belo N.O., Costa E.A., Andrade G.I., Pereira L.S., Carvalho I.A., Santos R.L. (2018). Frequency of enterotoxins, toxic shock syndrome toxin-1, and biofilm formation genes in *Staphylococcus aureus* isolates from cows with mastitis in the Northeast of Brazil. Trop. Anim. Health Prod..

[41] Attien P., Sina H., Moussaoui W., Zimmermann-Meisse G., Dadié T., Keller D., Riegel P., Edoh V., Kotchoni S.O., Djè M. (2014). Mass spectrometry and multiplex antigen assays to assess microbial quality and toxin production of *Staphylococcus aureus* strains isolated from clinical and food samples. BioMed Res. Int..

[42] Ogonowska P., Szymczak K., Empel J., Urbas M., Woźniak-Pawlikowska A., Barańska-Rybak W., Świetlik D., Nakonieczna J. (2023). *Staphylococcus aureus* from atopic dermatitis patients: Its genetic structure and susceptibility to phototreatment. Microbiol. Spectr..

[43] Taki Y., Watanabe S., Satoo Y., Tan X.-E., Ono H.K., Kiga K., Aiba Y., Sasahara T., Azam A.H., Thitiananpakorn K. (2022). The association between onset of Staphylococcal non-menstrual Toxic Shock Syndrome with inducibility of Toxic Shock Syndrome Toxin-1 production. Front. Microbiol..

[44] Li Y., Lei J., Qin X., Li G., Zhou Q., Yang Z. (2023). A mitochondria-targeted dual-response sensor for monitoring viscosity and peroxynitrite in living cells with distinct fluorescence signals. Bioinorg. Chem..

[45] Akineden Ö., Annemüller C., Hassan A.A., Lämmler C., Wolter W., Zschöck M. (2001). Toxin genes and other characteristics of *Staphylococcus aureus* isolates from milk of cows with mastitis. Clin. Diagn. Lab. Immunol..

[46] Katsuda K., Hata E., Kobayashi H., Kohmoto M., Kawashima K., Tsunemitsu H., Eguchi M. (2005). Molecular typing of *Staphylococcus aureus* isolated from bovine mastitic milk on the basis of toxin genes and coagulase gene polymorphisms. Vet. Microbiol..

[47] Jørgensen H.J., Mørk T., Høgåsen H.R., Rørvik L.M. (2005). Enterotoxigenic *Staphylococcus aureus* in bulk milk in Norway. J. Appl. Microbiol..

[48] Srinivasan V., Sawant A.A., Gillespie B.E., Headrick S.J., Ceasaris L., Oliver S.P. (2006). Prevalence of enterotoxin and toxic shock syndrome toxin genes in *Staphylococcus aureus* isolated from milk of cows with mastitis. Foodborne Path. Dis..

[49] Arcuri E.F., Angelo F.F., Guimaraes M.F.M., Talon R., de Fatima Borges M., Leroy S., Loiseau G., Lange C.C., de Andrade N.J., Montet D. (2010). Toxigenic status of *Staphylococcus aureus* isolated from bovine raw milk and Minas Frescal cheese in Brazil. J. Food Prot..

[50] Oliveira L., Rodrigues A.C., Hulland C., Ruegg P.L. (2011). Enterotoxin production, enterotoxin gene distribution, and genetic diversity of *Staphylococcus aureus* recovered from milk of cows with subclinical mastitis. Am. J. Vet. Res..

[51] Sur E., Turkyilmaz S. (2020). Investigation of the toxin genes and antibiotic resistance in *Staphylococcus aureus* isolates from subclinical mastitic cow milk. Israel J. Vet. Med..

[52] Kot B., Szweda P., Frankowska-Maciejewska A., Piechota M., Wolska K. (2016). Virulence gene profiles in *Staphylococcus aureus* isolated from cows with subclinical mastitis in eastern Poland. J. Dairy Res..

[53] Kang H.J., You J.Y., Kim S.H., Moon J.S., Kim H.Y., Kim J.M., Lee Y.J., Kang H.M. (2024). Characteristics of methicillin-resistant *Staphylococcus aureus* isolates from bovine mastitis milk in South Korea: Molecular characteristics, biofilm, virulence, and antimicrobial resistance. Microbiol. Spectr..

[54] Bonsaglia E.C.R., Silva N.C.C., Rossi B.F., Camargo C.H., Dantas S.T.A., Langoni H., Guimaraes F.F., Lima F.S., Fitzgerald J.R., Fernandes A. (2018). Molecular epidemiology of methicillin-susceptible *Staphylococcus aureus* (MSSA) isolated from milk of cows with subclinical mastitis. Microb. Pathog..

[55] Aguirre-Sánchez J.R., Campo N.C.D., Medrano-Félix J.A., Martínez-Torres A.O., Chaidez C., Querol-Audi J., Campo N.C.D. (2024). Genomic insights of *S. aureus* associated with bovine mastitis in a high livestock activity region of Mexico. J. Vet. Sci..

[56] Wodaje A., Belete M.A., Menkir A.S., Zegeye Z.B., Yihunie F.B. (2025). Detection of virulence genes and antimicrobial susceptibility profiles of *Staphylococcus aureus* isolates from bovine mastitis in Chagni, Northwestern Ethiopia. Vet. Med. Int..

[57] Orden J.A., Cid D., Blanco M.E., Ruiz Santa Quiteria J.A., Gomez-Lucia E., de la Fuente R. (1992). Enterotoxin and toxic shock syndrome toxin-one production by staphylococci isolated from mastitis in sheep. APMIS.

[58] Rahbarnia L., Khosravi R., Dehnad A.R., Naghili B. (2023). The examination of some virulence factors in *S. aureus* isolates obtained from the healthy human population, sheep mastitis, and cheese. Iran. J. Vet. Res. Shiraz Univ..

[59] Takeuchi S., Ishiguro K., Ikegami M., Kaidoh T., Hawakawa Y. (1996). Detection of toxic shock syndrome toxin-1 gene in *Staphylococcus aureus* bovine isolates and bulk milk by the polymerase chain reaction. J. Vet. Med. Sci..

[60] Murphy B.P., O’Mahony E., Buckley J.F., O’Brien S., Fanning S. (2010). Characterization of *Staphylococcus aureus* isolated from dairy animals in Ireland. Zoonoses Public Health.

[61] Giacinti G., Carfora V., Caprioli A., Sagrafoli D., Marri N., Giangolini G., Amoruso R., Iurescia M., Stravino F., Dottarelli S. (2017). Prevalence and characterization of methicillin-resistant *Staphylococcus aureus* carrying *mecA* or *mecC* and methicillin-susceptible *Staphylococcus aureus* in dairy sheep farms in central Italy. J. Dairy Sci..

[62] Vitale M., Scatassa M.L., Cardamone C., Oliveri G., Piraino C., Alduina R., Napoli C. (2015). Staphylococcal food poisoning case and molecular analysis of toxin genes in *Staphylococcus aureus* strains isolated from food in Sicily, Italy. Food Path. Dis..

[63] Soltan-Dallal M.M., Rajabi Z., Mirbagheri S.Z., Keyvanlou M., Didar Z. (2023). Characterization of antibiotic resistant *Staphylococcus* species and molecular identification of mecA and toxic shock syndrome toxin-1 (*tsst-1*) genes of *Staphylococcus aureus* isolates from cows’ milk. Int. Dairy J..

[64] Oliveira R., Pinho E., Almeida G., Azevedo N.F., Almeida C. (2022). Prevalence and diversity of *Staphylococcus aureus* and Staphylococcal enterotoxins in raw milk from northern Portugal. Front. Microbiol..

[65] Wisniewski P., Gajewska J., Zadernowska A., Chajecka P., Wierzchowska W. (2024). Identification of the enterotoxigenic potential of *Staphylococcus* spp. from raw milk and raw milk cheeses. Toxins.

[66] Tegegne H.A., Florianová M., Gelbíčová T., Karpíšková R., Koláčková I. (2019). Detection and molecular characterization of Methicillin-Resistant *Staphylococcus aureus* isolated from bulk tank milk of cows, sheep, and goats. Foodborne Path. Dis..

[67] Scherrer D., Corti S., Muehlherr J.E., Zweifel C., Stephan R. (2004). Phenotypic and genotypic characteristics of *Staphylococcus aureus* isolates from raw bulk-tank milk samples of goats and sheep. Vet. Microbiol..

[68] Mašlanková J., Pilipčincová I., Tkáčiková L. (2009). Pheno- and genotyping of *Staphylococcus aureus* isolates of sheep origin. Acta Vet. Brno.

[69] De Buyser M.L., Dilasser F., Hummel R., Bergdoll M.S. (1987). Enterotoxin and toxic shock syndrome toxin-1 production by staphylococci isolated from goat’s milk. Int. J. Food Microbiol..

[70] Valle J., Vadillo S., Piriz S., Gomez-Lucia E. (1991). Toxic shock syndrome toxin 1 (TSST-1) production by staphylococci isolated from goats and presence of specific antibodies to TSST-1 in serum and milk. Appl. Environ. Microbiol..

[71] Qian W., Shen L., Li X., Wang T., Liu M., Wang W., Fu Y., Zeng Q. (2019). Epidemiological Characteristics of *Staphylococcus aureus* in Raw Goat Milk in Shaanxi Province, China. Antibiotics.

[72] Liu H., Dong L., Zhao Y., Meng L., Wang J., Wang C., Zheng N. (2022). Antimicrobial susceptibility, and molecular characterization of *Staphylococcus aureus* isolated from different raw milk samples in China. Front. Microbiol..

[73] Akineden Ö., Hassan A.A., Schneider E., Usleber E. (2008). Enterotoxigenic properties of *Staphylococcus aureus* isolated from goats’ milk cheese. Int. J. Food Microbiol..

[74] Dai J., Wu S., Huang J., Wu Q., Zhang F., Zhang J., Wang J., Ding Y., Zhang S., Yang X. (2019). Prevalence and characterization of *Staphylococcus aureus* isolated from pasteurized milk in China. Front. Microbiol..

[75] Chaalal W., Chaalal N., Bourafa N., Kihal M., Diene S.D., Rolain J.M. (2018). Characterization of *Staphylococcus aureus* isolated from food products in Western Algeria. Foodborne Path. Dis..

[76] Salerno T., Siqueira A.K., Pinto J.P.A.N., da Cunha M.L.R.S., Silvestre P.K., Condas L.A.Z., Lara G.H.B., Pereira J.G., da Silva A.V., Listoni F.J.P. (2021). Safety issues of raw milk: Evaluation of bacteriological and physicochemical characteristics of human milk from a bank in a teaching hospital, focusing on *Staphylococcus* species. Rev. Inst. Med. Trop. Sao Paulo.

[77] Spanu V., Spanu C., Virdis S., Cossu F., Scarano C., de Santis E.P.L. (2012). Virulence factors and genetic variability of *Staphylococcus aureus* strains isolated from raw sheep’s milk cheese. Int. J. Food Microbiol..

[78] Aragao B.B., Trajano S.C., Silva J.G., Silva B.P., Oliveira R.P., Junior J.W.P., Peixoto R.M., Mota R.A. (2019). Short communication: High frequency of β-lactam-resistant *Staphylococcus aureus* in artisanal coalho cheese made from goat milk produced in northeastern Brazil. J. Dairy Sci..

[79] Minutillo R., Pirard B., Fatihi A., Cavaiuolo M., Lefebvre D., Gérard A., Taminiau B., Nia Y., Hennekinne J.-A., Daube G. (2023). The enterotoxin gene profiles and enterotoxin production of *Staphylococcus aureus* strains isolated from Artisanal Cheeses in Belgium. Foods.

[80] Mehli L., Hoel S., Merethe G., Thomassen B., Jakobsen A.N., Karlsen H. (2017). The prevalence, genetic diversity and antibiotic resistance of *Staphylococcus aureus* in milk, whey, and cheese from artisan farm dairies. Int. Dairy J..

[81] Sato T., Usui M., Konishi N., Kai A., Matsui H., Hanaki H., Tamura Y. (2017). Closely related methicillin-resistant *Staphylococcus aureus* isolates from retail meat, cows with mastitis, and humans in Japan. PLoS ONE.

[82] Chairat S., Haythem G., Carmen L., Gómez-Sanz E., Zarazaga M., Abdellatif B., Torres C., Ben Slama K. (2015). Characterization of *Staphylococcus aureus* from Raw Meat Samples in Tunisia: Detection of Clonal Lineage ST398 from the African Continent. Foodborne Path. Dis..

[83] Abdalrahman L.S., Wells H., Fakhr M.K. (2015). *Staphylococcus aureus* is more prevalent in retail beef livers than in pork and other beef cuts. Pathogens.

[84] Mahros M.A., Abd-Elghany S.M., Sallam K.I. (2021). Multidrug-, methicillin-, and vancomycin-resistant *Staphylococcus aureus* isolated from ready-to-eat meat sandwiches: An ongoing food and public health concern. Int. J. Food Microb..

[85] Dawood R.M., Rasha M., Alsanjary R.A. (2025). Molecular detection of some Staphylococcal enterotoxins in the meat of slaughtered animals in abattoirs, Mosul-Iraq. Iraqi J. Vet. Sci..

[86] Sapugahawatte D.N., Li C., Yeoh Y.K., Dharmaratne P., Zhu C., Ip M. (2020). Swine methicillin-resistant *Staphylococcus aureus* carrying toxic-shock syndrome toxin gene in Hong Kong, China. Emerg. Microbes Infect..

[87] Arfatahery N., Davoodabadi A., Abedimohtasab T. (2016). Characterization of toxin genes and antimicrobial susceptibility of *Staphylococcus aureus* isolates in fishery products in Iran. Sci. Rep..

[88] Rong D., Wu Q., Xu M., Zhang J., Yu S. (2017). Prevalence, virulence genes, antimicrobial susceptibility, and genetic diversity of *Staphylococcus aureus* from retail aquatic products in China. Front. Microbiol..

[89] Dai J., Huang J., Wu S., Zhang F., Li Y., Rong D., Zhao M., Ye Q., Gu Q., Zhang Y. (2023). Occurrence, antibiotic susceptibility, biofilm formation and molecular characterization of *Staphylococcus aureus* isolated from raw shrimp in China. Foods.

[90] Mohammed R., Nader S.M., Hamza D.A., Sabry M.A. (2025). Public health implications of multidrug resistant and methicillin resistant *Staphylococcus aureus* in retail oysters. Sci. Rep..

[91] Hammad A.M., Wataru W., Tomoko T., Shimamoto T. (2012). Occurrence and characteristics of methicillin-resistant and –susceptible *Staphylococcus aureus* and methicillin-resistant coagulase-negative staphylococci from Japanese retail ready-to-eat raw fish. Int. J. Food Microb..

[92] Oh S.K., Lee N., Cho Y.S., Shin D.B., Choi S.Y., Koo M. (2007). Occurrence of toxigenic *Staphylococcus aureus* in ready-to-eat food in Korea. J. Food Prot..

[93] Mekhloufi O.A., Chieffi D., Hammoudi A., Bensefia S.A., Fanelli F., Fusco V. (2021). Prevalence, enterotoxigenic potential and antimicrobial resistance of *Staphylococcus aureus* and Methicillin-Resistant *Staphylococcus aureus* (MRSA) isolated from Algerian ready to eat foods. Toxins.

[94] Elsalkh S.A., Zakaria A.I., Abd-Elghany S.M., Imre K., Morar A., Sallam K.I. (2025). Prevalence and characterization of the antimicrobial resistance and virulence profiles of *Staphylococcus aureus* in ready-to-eat (meat, chicken, and tuna) pizzas in Mansoura City, Egypt. Antibiotics.

[95] Ballah F.M., Islam M.S., Rana M.L., Ullah M.A., Ferdous F.B., Neloy F.H., Ievy S., Sobur M.A., Rahman A.M.M.T., Khatun M.M. (2022). Virulence determinants and methicillin resistance in biofilm-forming *Staphylococcus aureus* from various food sources in Bangladesh. Antibiotics.

[96] Soltan-Dallal M.M., Salehipour Z., Eshraghi S., Mehrabadi J.F., Bakhtiari R. (2010). Occurrence and molecular characterization of *Staphylococcus aureus* strains isolated from meat and dairy products by PCR-RFLP. Ann. Microbiol..

[97] Alibayov B., Zdenkova K., Sykorova H., Demnerova K. (2014). Molecular analysis of *Staphylococcus aureus* pathogenicity islands (SaPI) and their superantigens combination of food samples. J. Microb. Meth..

[98] Adame-Gómez R., Castro-Alarcón N., Vences-Velázquez A., Toribio-Jiménez J., Pérez-Valdespino A., Leyva-Vázquez M.A., Ramírez-Peralta A. (2020). Genetic diversity and virulence factors of *S. aureus* isolated from food, humans, and animals. Int. J. Microbiol..

[99] Liang T., Liang Z., Wu S., Ding Y., Wu Q., Gu B. (2023). Global prevalence of *Staphylococcus aureus* in food products and its relationship with the occurrence and development of diabetes mellitus. Med. Adv..

[100] Baniardalan S., Mohammadzadeh A., Pajohi-Alamoti M., Mahmoodi P., Sadeghinasab A. (2017). Detection of toxic shock toxin (*tst*) gene in *Staphylococcus aureus* isolated from bovine milk samples. Bulg. J. Vet. Med..

[101] Léguillier V., Pinamonti D., Chang C., Gunjan R., Mukherjee R., Himanshu A., Cossetini A., Manzano M., Anba-Mondoloni J., Malet-Villemagne J. (2024). A review and meta-analysis of *Staphylococcus aureus* prevalence in foods. Microbe.

[102] Ou Q., Zhou J., Lin D., Bai C., Zhang T., Lin J., Zheng H., Wang X., Ye J., Ye X. (2018). A large meta-analysis of the global prevalence rates of *S. aureus* and MRSA contamination of milk. Crit. Rev. Food Sci. Nutr..

[103] Kerro Dego O., Vidlund J. (2024). Staphylococcal mastitis in dairy cows. Front. Vet. Sci..

[104] Rainard P., Foucras G., Fitzgerald J.R., Watts J.L., Koop G., Middleton J.R. (2018). Knowledge gaps and research priorities in *Staphylococcus aureus* mastitis control. Transbound. Emerg. Dis..

[105] Imanishi I., Nicolas A., Caetano A.C.B., de Castro T.L.P., Tartaglia N.R., Mariutti R., Guédon E., Even S., Berkova N., Arni R.K. (2019). Exfoliative toxin E, a new *Staphylococcus aureus* virulence factor with host-specific activity. Sci. Rep..

[106] Artursson K., Söderlund R., Liu L., Monecke S., Schelin J. (2016). Genotyping of *Staphylococcus aureus* in bovine mastitis and correlation to phenotypic characteristics. Vet. Microbiol..

[107] Yang A., Ye Y., Liu Q., Xu J., Li R., Xu M., Wang X., Fu S., Yu R. (2025). Response of Nutritional Values and Gut Microbiomes to Dietary Intake of ω-3 Polyunsaturated Fatty Acids in *Tenebrio molitor* Larvae. Insects.

[108] Pexara A., Solomakos N., Govaris A. (2020). Occurrence, antibiotic resistance and enteroxigenicity of *Staphylococcus* spp. in tonsils of slaughtered pigs in Greece. Lett. Appl. Microbiol..

[109] Sarkar S. (2015). Microbiological considerations: Pasteurized milk. Int. J. Dairy Sci..

[110] Lin Y., Pan M., Yang Y., Ye X., Xu H., Lin Y., Niu B. (2025). Exposure assessment of *Staphylococcus aureus* in fluid milk. Int. Dairy J..

[111] Zhang L., Jin Y., Xue L., Zhang H., Yang N., Xu X. (2025). Inactivation effects on *Escherichia coli* in selected liquid food models by induced electric field: Germicidal efficacy and putative mechanism. Food Bioprocess. Technol..

[112] Xue L., Yang N., Xu X., Jin Y., Cao X., Zhang H. (2025). Inactivation of *Geobacillus stearothermophilus* spores by induced electric field in different food mediums. Food Microb..

[113] Chen Z., Song Y., Yan Y., Chen W., Ren T., Ma A., Li S., Jia Y. (2025). Characterization of an epilactose-producing cellobiose 2-epimerase from *Clostridium* sp. TW13 and reutilization of waste milk. Food Chem..

[114] Rabbani A., Ayyash M., D’Costa C.D.C., Chen G., Xu Y., Kamal-Eldin A. (2025). Effect of heat pasteurization and sterilization on milk safety, composition, sensory properties, and nutritional quality. Foods.

[115] Fusco V., Chieffi D., Fanelli F., Logrieco A.F., Cho G.S., Kabisch J., Böhnlein C., Franz C.M.A.P. (2020). Microbial Quality and Safety of Milk and Milk Products in the 21st Century. Compr. Rev. Food Sci. Food Saf..

[116] Silva G.O.E., Gabriela Oliveira E.R.D., Castro R.D., Oliveira L.G., Sant’Anna F.M., Machado F., Barbosa C.D., Cosme Damião C., Sandes S.H.D.C., Sávio Henrique De Cicco R.S. (2020). Viability of *Staphylococcus aureus* and expression of its toxins (SEC and TSST-1) in cheeses using *Lactobacillus rhamnosus* D1 or *Weissella paramesenteroides* GIR16L4 or both as starter cultures. J. Dairy Sci..

[117] Zhang J., Wang J., Jin J., Li X., Zhang H., Shi X., Zhao C. (2022). Prevalence, antibiotic resistance, and enterotoxin genes of *Staphylococcus aureus* isolated from milk and dairy products worldwide: A systematic review and meta-analysis. Food Res. Int..

[118] Ogata K., Narimatsu H., Suzuki M., Higuchi W., Yamamoto T., Taniguchi H. (2012). Commercially distributed meat as a potential vehicle for community-acquired methicillin-resistant *Staphylococcus aureus*. Appl. Environ. Microbiol..

[119] Mørk T., Kvitle B., Jørgensen H.J. (2012). Reservoirs of *Staphylococcus aureus* in meat sheep and dairy cattle. Vet. Microb..

[120] Concepción Porrero M., Hasman H., Vela A.I., Fernandez-Garayzabal J.F., Domínguez L., Aarestrup F.M. (2012). Clonal diversity of *Staphylococcus aureus* originating from the small ruminants goats and sheep. Vet. Microb..

[121] Boss R., Cosandey A., Luini M., Artursson K., Bardiau M., Breitenwieser F., Hehenberger E., Lam T., Mansfeld M., Michel A. (2016). Bovine *Staphylococcus aureus*: Subtyping, evolution, and zoonotic transfer. J. Dairy Sci..

[122] Pexara A., Solomakos N., Sergelidis D., Govaris A. (2012). Fate of enterotoxigenic *Staphylococcus aureus* and staphylococcal enterotoxins in Feta and Galotyri cheeses. J. Dairy Res..

[123] Monecke S.A., Ruppelt S., Wendlandt S., Schwarz P., Slickers R., Ehricht R., Jäckel S.C. (2013). Genotyping of *Staphylococcus aureus* isolates from diseased poultry. Vet. Microbiol..

[124] Kim Y.B., Seo K.W., Jeon H.Y., Lim S.K., Lee Y.L. (2018). Characteristics of the antimicrobial resistance of *Staphylococcus aureus* isolated from chicken meat produced by different integrated broiler operations in Korea. Poultry Sci..

[125] Thwala T., Madoroba E., Basson A., Butaye P. (2021). Prevalence and characteristics of *Staphylococcus aureus* associated with meat and meat products in African countries: A Review. Antibiotics.

[126] Zhu Z., Liu X., Chen X., Zou G., Huang Q., Meng X., Pei X., Chen Z., Zhou R., Hu D. (2022). Prevalence and virulence determinants of *Staphylococcus aureus* in wholesale and retail pork in Wuhan, central China. Foods.

[127] Sanlıbaba P. (2022). Prevalence, antibiotic resistance, and enterotoxin production of *Staphylococcus aureus* isolated from retail raw beef, sheep, and lamb meat in Turkey. Int. J. Food Microb..

[128] Li S., Wang P., Zhao J., Zhou L., Zhang P., Fu C., Meng J., Wang X. (2018). Characterization of toxin genes and antimicrobial susceptibility of *Staphylococcus aureus* from retail raw chicken meat. J. Food Prot..

[129] Sivaraman G.K., Gupta S.S., Visnuvinayagam S., Muthulakshmi T., Elangovan R., Perumal V., Balasubramanium G., Lodha T., Yadav A.V. (2022). Prevalence of *S. aureus* and/or MRSA from seafood products from Indian seafood products. BMC Microb..

[130] Aguirre-Sánchez J.R., Chaidez-Quiroz C., Castro-del Campo N. (2025). Genetic characterization of *Staphylococcus aureus* isolates associated with Toxic Shock Syndrome toxin production: An epidemiological and bioinformatics approach. Toxins.

[131] Yang X., Yu S., Wu Q., Zhang J., Wu S., Rong D. (2018). Multilocus sequence typing and virulence-associated gene profile analysis of *Staphylococcus aureus* isolates from retail ready-to-eat food in China. Front. Microbiol..

[132] Jain M., Parida A., Rani V., Gaind R. (2025). Virulence genes encoding for Panton Valentine Leucocidin and Toxic Shock Syndrome Toxin in methicillin resistant *Staphylococcus aureus*. J. Med. Bacteriol..

[133] Babic M., Pajić M., Nikolić A., Teodorović V., Mirilović M., Milojević L., Velebit B. (2018). Expression of toxic shock syndrome toxin-1 gene of *Staphylococcus aureus* in milk: Proof of concept. *Staphylococcus aureus* in milk. Mljekarstvo.

[134] Li S.J., Hu D.L., Maina E.K., Shinagawa K., Omoe K., Nakane A. (2011). Superantigenic activity of toxic shock syndrome toxin-1 is resistant to heating and digestive enzymes. J. Appl. Microb..

[135] He Y., Zhang P., Wang X., Wang Z., Xu S., Liu J., Jia C., Cai S., Yue Z., Zhang H. (2025). Pre-loaded H_2_O_2_ breaks the low-pH dependency of plasma-activated water for bacteria inactivation via catalase suppression and oxidative attack. Appl. Phys. Lett..

[136] Argudín M.A., Mendoza M.C., Rodicio M.R. (2010). Food Poisoning and *Staphylococcus aureus* Enterotoxins. Toxins.

[137] The Commission of the European Communities (2005). Commission Regulation (EC) No 2073/2005 of 15 November 2005 on Microbiological Criteria for Foodstuffs (Text with EEA Relevance).

[138] Chiang Y.C., Liao W.W., Fan C.M., Pai W.Y., Chiou C.S., Tsen H.Y. (2008). PCR detection of Staphylococcal enterotoxins (SEs) N, O, P, Q, R, U, and survey of SE types in *Staphylococcus aureus* isolates from food-poisoning cases in Taiwan. Int. J. Food Microb..

[139] Goudsmit A., Markowicz S., Lali S.E., Cherifi S. (2021). Food poisoning due to a TSST1-producing *Staphylococcus aureus*. IDCases.

[140] Cha J.O., Lee J.K., Jung Y.H., Yoo J.I., Park Y.K., Kim B.S., Lee Y.S. (2006). Molecular analysis of *Staphylococcus aureus* isolates associated with staphylococcal food poisoning in South Korea. J. Appl. Microbiol..

